# Validating a Bayesian Spatio-Temporal Model to Predict La Crosse Virus Human Incidence in the Appalachian Mountain Region, USA

**DOI:** 10.3390/microorganisms13040812

**Published:** 2025-04-03

**Authors:** Maggie McCarter, Stella C. W. Self, Huixuan Li, Joseph A. Ewing, Lídia Gual-Gonzalez, Mufaro Kanyangarara, Melissa S. Nolan

**Affiliations:** 1 Department of Epidemiology and Biostatistics, Arnold School of Public Health, University of South Carolina, 915 Greene Street, Columbia, SC 29208, USA; maggiemccarter@gmail.com (M.M.); scwatson@mailbox.sc.edu (S.C.W.S.); huixuan@email.sc.edu (H.L.); lidiag@email.sc.edu (L.G.-G.); mufaro@mailbox.sc.edu (M.K.); 2 Data Support Core, Prisma Health, 701 Grove Rd, Greenville, SC 29605, USA; alex.ewing@prismahealth.org

**Keywords:** arboviral surveillance, forecast modelling, prediction modelling, integrated nested laplace approximation technique, bayesian statistics

## Abstract

La Crosse virus (LACV) is a rare cause of pediatric encephalitis, yet identifying and mitigating transmission foci is critical to detecting additional cases. Neurologic disease disproportionately occurs among children, and survivors often experience substantial, life-altering chronic disability. Despite its severe clinical impact, public health resources to detect and mitigate transmission are lacking. This study aimed to design a Bayesian modelling approach to effectively identify and predict LACV incidence for geospatially informed public health interventions. A Bayesian negative binomial spatio-temporal regression model best fit the data and demonstrated high accuracy. Nine variables were statistically significant in predicting LACV incidence for the Appalachian Mountain Region. Proportion of children, proportion of developed open space, and proportion of barren land were positively associated with LACV incidence, while vapor pressure deficit index, year, and proportions of developed high intensity land, evergreen forest, hay pasture, and woody wetland were negatively associated with LACV incidence. Model prediction error was low, less than 2%, indicating high accuracy in predicting annual LACV human incidence at the county level. In summary, this study demonstrates the utility of Bayesian negative binomial spatio-temporal regression models for predicting rare but medically important LACV human cases. Future studies could examine more granular models for predicting LACV cases from localized variables such as mosquito control efforts, local reservoir host density and local weather fluctuations.

## 1. Introduction

La Crosse virus (LACV) is a mosquito-borne disease endemic in the Appalachian region of the United States of America. This California serogroup encephalitis virus was first identified in 1965 in the brain and spinal tissue of a fatal pediatric encephalitis case in La Crosse, Wisconsin [1]. This virus, named after the city, is now the second most commonly reported arbovirus in the USA, behind West Nile virus [2]. Currently, up to 150 LACV cases are reported in the USA each year. However, as less severe cases of LACV are often misdiagnosed and underreported, there is likely higher disease prevalence [3]. Historical studies have shown that the true prevalence is potentially much greater, with yearly incidence estimates being as high as 300,000 cases in the USA [3,4,5,6].

LACV is more commonly diagnosed in children, and survivors of neuroinvasive disease experience substantial sequalae and permanent disability—devastating consequences considering LACV cases are eight years old on average [7]. Pediatric survivors frequently face long-term disabilities such as reduced cognitive function, attention deficit disorders, and seizures [8,9]. Adult survivors of severe LACV infections can also experience sequalae such as seizures and changes in mental state [10]. There are serious economic implications as well, as neurological damages resulting from LACV infection can have a financial impact ranging from USD 7000 to USD 3 million per case [11].

La Crosse virus cases are primarily reported in the Midwest and Appalachia regions of the USA, although recently LACV has been detected in mosquitos as far north as New England [12]. LACV is most prevalent in areas where hardwood forests are predominant, providing the ideal environment for its primary vector: *Aedes triseriatus* (Say), the North American tree hole mosquito [13]. These mosquitos breed inside water-filled holes created when heavy hardwood branches break off during tree growth and maturation. Mosquito vectors can transmit the virus through transovarial, transstadial, and venereal mechanisms, allowing for sustained multi-generational focal transmission risk areas [14]. These vector biology mechanisms allow LACV positive mosquito populations to survive through the winter and contribute to recurrent disease transmission [13,15]. While *Aedes triseriatus* is the primary vector for LACV, three other secondary vectors may contribute to human transmission: *Aedes albopictus* (Skuse)*, Aedes japonicus* (Theobald), and *Aedes aegypti* (L.) [13,16]. Virus transmission is amplified through rodents, mainly chipmunks and grey squirrels, which serve as an infection source for mosquitoes transmitting the virus to humans. Humans are dead-end hosts, as the virus cannot be transmitted from human to human [17,18,19].

Surveillance and preventative efforts to control LACV in the Eastern USA are scarce and often limited to sparse vector surveillance and resource-constrained investigations [20,21]. Studies have linked Eastern chipmunk (*Tamias striatus*) population density, rural residence, and deciduous forest cover to increased hotspots of LACV incidence [14,18]. Additionally, lower socioeconomic status and high proportions of children living in an area have been found to predict LACV incidence [9,18,22]. Recently, states within the Appalachian Mountain region have begun mosquito surveillance programs to better inform policymakers and the community [23,24]; but given the time-consuming and resource-dependent nature of such programs, the incorporation of mathematical LACV incidence forecasting could strengthen these initiatives.

With the advancement of mathematical and statistical modeling, infectious disease forecast modeling techniques have frequently been used by governments and institutions to influence public health decisions and initiatives [25]. Bayesian spatio-temporal regression models have been heavily used in arboviral forecasts [26,27]. Historically, Markov chain Monte Carlo (MCMC) methods have been used to fit such models [28,29]. However, MCMC can be computationally expensive. To mitigate this, statisticians have established alternative methods for fitting these models, such as the integrated nested Laplace approximation (INLA) method [30], that provides an accurate yet computationally efficient means of disease forecasting. Despite these advancements, no studies have applied such techniques for the modeling and forecasting of human LACV cases in the USA. Therefore, this study aims to demonstrate the utility of a Bayesian negative binomial regression model to predict county-level human LACV incidence.

## 2. Materials and Methods

### 2.1. La Crosse Virus Reported Human Incidence Data for the Eastern USA and Appalachian Mountain Region

LACV human cases are a nationally notifiable disease. The U.S. Centers for Disease Control and Prevention (CDC) aggregates reported human case counts by county and year; these data are publicly available on the CDC’s ArboNET database website [31]. This database maintains over 20 years of national county-level data on arboviral infections in humans, animals, and vectors. For this research study, data were selected from 408 counties served by the Appalachian Regional Commission (ARC) for the years 2010–2022.

### 2.2. Covariates

Covariates were selected through expert interviews (medical entomologist, vector-borne disease epidemiologist, and biostatistician) and a thorough literature search. Because LACV is the most common neuroinvasive arboviral infection in children, we obtained the percent pediatric population per county per year from the USA Census Bureau [8]. Yearly rodent density data, including the Eastern chipmunk and Eastern grey squirrel (*Sciurus carolinensis*), for the years 2008–2016 were available and obtained from the United States Geological Survey [32,33]. Land cover data, including landcover and land use status, were retrieved from the National Land Cover Database (NLCD) for the years 2001, 2006, 2011, and 2019 [34]. Linear interpolation and extrapolation were performed to estimate land cover for years when NLCD data were not available. Weather data for precipitation, temperature, and humidity were obtained at the county level and averaged on a yearly basis from the Northwest Alliance for Computational Science’s PRISM climate dataset [35].

### 2.3. The Model

We used a Bayesian negative binomial regression model, which is described by the following equations:

(1) $$Y_{st}|\mu_{st},\;\sigma^{2}\sim NB(\mu_{st},\;\sigma^{2})$$

(2) $$\mu_{st}=exp(\eta_{st})$$

(3) $$\eta_{st}=\beta_{0}+\vec{\beta }\;{\vec{x}}_{st}+\phi_{st}.$$

The number of cases in county *s* and year *t*, $Y_{st}$ follows a negative binomial distribution with mean $\mu_{st}$; as usual, $\mu_{st}$ is related to a linear predictor $\eta_{st}$, a function of covariates, through the log link function. The negative binomial distribution also includes an overdispersion parameter, denoted here by $\sigma^{2}.$ Three pieces compose $\eta_{st}$: $\beta_{0}$, a shared intercept term, covariate factors ${\vec{x}}_{st}$, and an offset term $\phi_{st}$, equal to the log of the population of county *s* and year *t*.

### 2.4. Model Selection

Before model selection, multicollinearity among the variables was assessed using variance inflation factors (VIFs). Any variables with a VIF greater than five were removed from analysis until all remaining variables had VIF values below five. Rodent density data for grey squirrels and Eastern chipmunks were excluded due to the lack of spatial or temporal variation. The following variables were removed: proportion of developed medium intensity, developed low intensity, deciduous forest landcover categories, and mean temperature. All continuous variables were standardized prior to model fitting. The full model included the proportion of children aged 19 and under, open water, developed open space, developed high intensity (urban), barren land, evergreen forest, mixed forest, shrub scrub, herbaceous, hay pasture, and woody wetlands landcover, average precipitation, vapor pressure deficit (VPD) index, percent below the poverty line, and a year variable. After removing variables exhibiting multicollinearity, spatial autocorrelation was assessed by applying the global Moran’s I statistic on the full model residuals. No spatial autocorrelation in the residuals was detected, so spatially varying random effects were not included. Additionally, the model was assessed for temporal autocorrelation via autocorrelation function (ACF) plots [36,37]. We fit the full model, which included a linear fixed effect for time but no random effects, and assessed each ACF plot. Temporal autocorrelation was not found; therefore, temporal random effects were not included.

After the autocorrelation assessment, model selection was performed. Beginning with the full model, variables whose 95% credible interval contained ‘0’ were considered for removal one at a time. However, to facilitate mathematical forecasting, the year variable was not eligible for removal. The standardized absolute value, defined as the absolute value of the posterior mean divided by the posterior standard deviation, was computed for each statistically non-important variable. We then identified the non-important variable with the smallest standardized absolute value, removed it, and refit the model. This was repeated until only statistically important variables remained in the model. An INLA approach was used to fit the models, introduced by Rue et al. (2009) [30]. Such approximation allows for an accurate yet computationally efficient means of fitting Bayesian models, much faster than a standard MCMC method. All map figures and analyses were performed using R studio software (version 22.12.0).

### 2.5. Modeling Prediction and Inter-Year Accuracy

To assess model prediction accuracy, data from each year (2010–2021) were withheld one at a time. The model was fit using data from all other years, and LACV cases were predicted for the withheld year. This leave-one-out validation process resulted in 12 distinct models. Predicted values were compared to the actual data using the mean square prediction error. Additionally, ‘forecast’ predictions were made for the years 2015–2021 using data up to but not including each forecast year. The first strategy assessed the model’s ability to predict cases when data is observed both before and after the point of prediction; the second strategy assessed the model’s ability to forecast future cases from past data alone.

## 3. Results

Model validation was performed using two strategies: leave-one-out predictions for the years 2010–2021 and standard predictions for the years 2015–2021. The posterior mean estimates, standard deviations, and 95% highest posterior density intervals for all years can be found in Table 1. The mean square prediction error for each year of the leave-one-out predictions, in addition to the counties with error greater than two counts, can be found in Table S1. These leave-one-out models performed well, with a maximum of only 2.28% of counties having errors above two counts. The mean square prediction error and counties with the largest error for the traditional prediction validation can be found in Table S2. The traditional prediction models also performed well, with a maximum of 1.95% of counties with an error greater than two counts.

**Figure 1:**
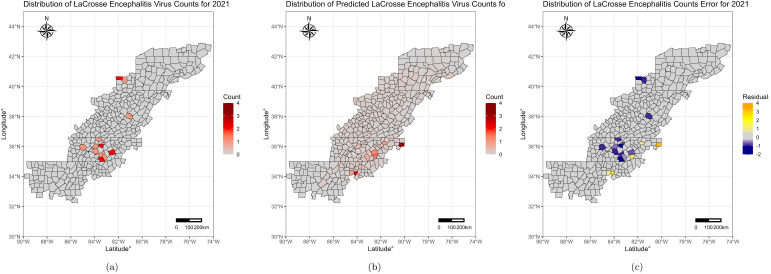
Reported, Predicted and Model Error for La Crosse Virus Human Counts, 2021. (**a**) Actual counts of reported human LACV incident cases in 2021 by county; (**b**) INLA model predicted LACV incident human case counts for the same year; (**c**) County-level count errors between actual vs. predicted LACV incidence.

Figure 1a displays the observed LACV counts for the year 2021: Macon, NC, Holmes, OH, Jefferson, TN, and Buncombe, NC, which all had the highest counts (>two cases). Figure 1b exhibits the predicted LACV counts for the same year: Forsyth, NC and Forsyth, GA counties were predicted to have the highest counts. The distribution of the 95% highest posterior distribution lower and upper bounds can be seen in Figure S1. Finally, Figure 1c shows the error residuals between actual versus predicted LACV human case counts for 2021. Minimal error was noted, with residuals ranging from −1.85 to 3.24; Forsyth, NC, and Forsyth, GA displayed the largest residual errors (3.24 and 2.51 counts, respectively).

**Figure 2:**
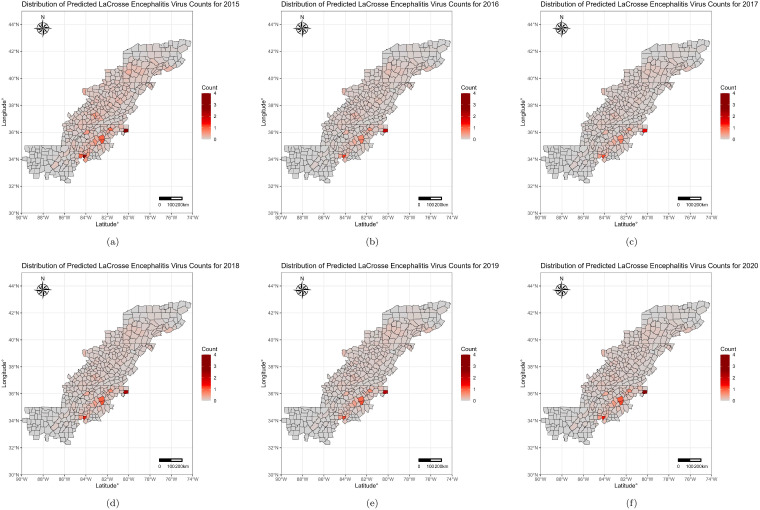
Yearly INLA-Predicted La Crosse virus Human Counts by County for the Years 2015–2020. INLA model predicted LACV incident human case counts for (**a**) 2015; (**b**) 2016; (**c**) 2017; (**d**) 2018; (**e**) 2019; and (**f**) 2020.

**Figure 3:**
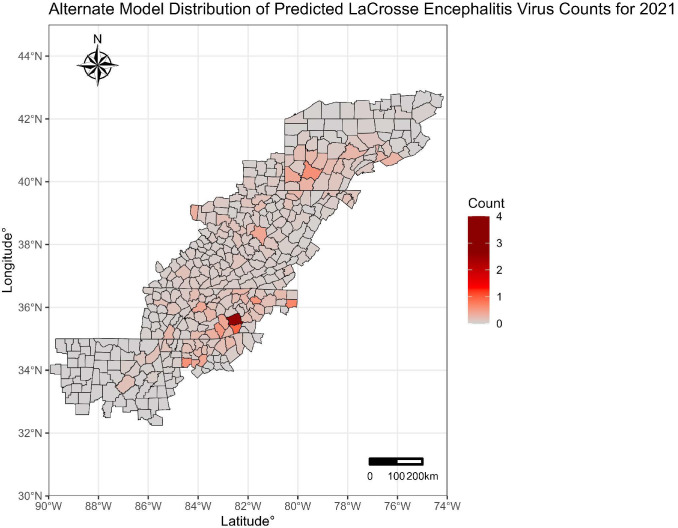
Alternate Poisson Model-Predicted Case Counts, 2021.

A spatial pattern was evident in human case counts for the years 2015–2020 for the traditional forecasting models as shown in Figure 2. Across all the years, Forsyth, NC and Forsyth, GA were consistently predicted to have the highest LACV counts. An alternative selection analysis was performed with a Poisson regression model, using a similar variable elimination process. As noted in Figure 3, this alternative model demonstrated subpar prediction, with only one county predicted to have one or more LACV incident cases.

## 4. Discussion

Despite the clinical and economic impact of LACV, the resources allocated to disease prevention are still very limited, and the existing programs receive little funding. Initiatives to mitigate the *Aedes triseriatus* mosquito by limiting potential larval habitats have proven useful, but more research is needed to better develop targeted approaches in high-risk areas [38,39]. Forecasting models have proven useful in informing regional stakeholders to target prevention efforts for other arboviruses such as West Nile virus and dengue virus [26,27,40]. The utilization of arboviral forecasting is an imperative step in reducing La Crosse encephalitis virus transmission, particularly in the Appalachian region.

The model performed well in predicting LACV cases and incidence across the Appalachian region. The maximum absolute prediction error for the year 2021 was 3.24 cases. Across all years, the model accurately predicted cases in up to 98.08% of Appalachian counties with errors fewer than two counts. However, the model consistently over-predicted cases in Forsyth, NC and Forsyth, GA in both the traditional forecasting and leave-one-out approaches. Further exploration of these counties was performed to assess potential influences both counties have on prediction, though the analysis did not reveal similarities that might explain these patterns. However, this could be due to effects not captured by the data such as reporting biases or ecological or behavioral factors (e.g., unusually high human–mosquito contact). Additionally, the model underpredicted cases in counties that experienced the highest counts of LACV each year across all leave-one-out and traditional forecasting years. Acknowledging the consistent under and overprediction in these counties can serve as a guide to increase efforts to better inform the model and improve the accuracy of the forecasting.

It should be noted that the value in these predictions lies in not only the predicted case counts, but the predicted incidence patterns. Specifically, as LACV infection in counties only ranged by a few counts, institutions interested in assessing the pattern of LACV spread should focus on the spatial patterns of predicted incidence. A distinct pattern of increased incidence was observed in the midwestern part of the Appalachian region. Following such incidence patterns could serve to prepare governments and public health authorities to increase surveillance and prepare for higher number of LACV cases. Efforts, such as larval control and public education campaigns, would especially benefit from knowledge of disease spread.

Some findings of demographic, landscape, and climate factors influencing LACV incidence from the 2021 prediction model align with previous studies. For instance, the proportion of children aged 19 and under was positively associated with LACV incidence. This coincides with Vahey et al. (2021) who found the median age of LACV incidence in the years 2003–2019 to be eight years old, with 88% of cases being younger than 18 years old [7]. Similarly, developed high intensity areas (cityscape) and hay pasture were negatively associated with LACV incidence—this can be expected as the primary host of LACV, *Aedes triseriatus*, commonly resides in hardwood forests; as it prefers ovipositing in abscesses created by rotting and falling limbs [13]. In contrast, barren land was positively associated with higher LACV rates, which coincides with work by Laporta et al. (2021) who found barren land segments in habitats, particularly due to deforestation, increased rates of mosquito-borne diseases [41]. Deforestation has been a significant challenge in Appalachia, making barren land susceptible to erosion [42]. Additionally, barren land also reflects areas of human disturbance and urbanization, which has been shown to lead to increased larval habitats such as artificial containers [43]. Although efforts are being made to mitigate this, it holds the potential consequence of increased LACV incidence.

Additionally, developed open space—commonly parks, golf courses, and recreational vegetation—was positively associated with increased LACV rates, which is consistent with the existing literature on mosquito populations within suburban landscapes [44]. Additionally, *Aedes japonicus*, a secondary vector of LACV, is known to inhabit suburban landscapes in areas such as rock pools and artificial water features [45,46]. Vapor pressure deficit (VPD) index, the difference between the recorded moisture in the air and how much moisture saturated air can hold, was found to be negatively associated with LACV incidence. VPD is inversely related to humidity and therefore this finding supports the existing literature establishing a positive association between humidity and LACV [47]. However, there is a bell-shaped curve of the relationship between humidity and mosquito population, in which very high concentrations of humidity result in smaller mosquito populations [48]. However, research has shown that there is a potential lag-time regarding the effect of climactic factors such as VPD and humidity and arboviruses [49]. Future research could benefit from taking this lag-time into account when considering these factors. Additionally, *Aedes japonicus* mosquitos thrive in temperate climates, and *Aedes triseriatus* mosquitos have adapted to lower temperatures through diapause; both may have adapted to areas with lower VPD to avoid competition with other mosquito vectors adapted to warmer regions.

The model revealed some significant variables contradictory to the published literature. LACV has been shown to disproportionately affect individuals with low socioeconomic status [22]. However, the percentage of the population below the poverty line was not found to be statistically important in our model. This finding could be explained by the limited access to healthcare experienced by many low socioeconomic status individuals: marginalized individuals less frequently seek medical care, and thus cases may be underreported. Additionally, socioeconomic status could be confounded by rurality, which could independently drive LACV incidence through environmental factors. The West Virginia Appalachian region hosts the ideal environment for LACV vectors, making LACV the most common arboviral infection in the state [50]. However, the model did not predict many cases in the state for 2021, nor were there a substantial number of cases reported in 2021. This reflects the study’s sampling bias (using reported case data) and exemplifies the value of multi-dataset modelling to help unearth areas of potential surveillance neglect. Like any model of an underreported disease, the model predicts the number of reported LACV cases, which is likely an underestimate of the true number of cases. Unless LACV surveillance improves enough to detect every case, the degree of disparity between the number of cases predicted by the model and the true number of cases will remain impossible to precisely quantify.

Associations between environmental variables and LACV counts are particularly useful in the context of climate change. As the landscape and weather continue to change, relationships between these environmental factors and LACV incidence can be used to identify areas in which the risk of LACV may be increasing. While our model did not assess the causality of these relationships, it did demonstrate that certain landscape and weather variables are strongly predictive of LACV counts. Given the rarity of LACV, informing medical providers of increasing risk in their areas may aid in the timely diagnosis and treatment of LACV, potentially reducing the public health burden of this arbovirus.

Although the model accurately predicted LACV counts and incidence within the Appalachian region, it is not without limitations. Arboviral infections are frequently underreported and underdiagnosed, especially in lower-income rural areas highly present in the Appalachian region [51]. Because of this, LACV counts are likely underestimated in this study, as consistently lower reported numbers result in low predicted numbers for the following years. The Eastern chipmunk and the Eastern gray squirrel are two key LACV amplifying hosts that were hoped to be incorporated into the model; however, the available United States Geological Survey dataset reported these species in every Appalachian region county without further granularity, and therefore the influence of these amplifying hosts could not be meaningfully analyzed in the model. Some spatial dependence is evident in the residuals from the final model. Ideally, this could be adjusted by including a spatial random effect; however, spatial and independent random effects caused the model to fail to converge and were therefore excluded from the model. Future studies could implement methods such as geographically weighted regression to potentially address this. It should be noted that a zero-inflated negative binomial regression was considered, but it performed poorly relative to the standard negative binomial. As the overall mean count in the data is low, it is reasonable to assume the zeros arise from the negative binomial distribution itself rather than from a separate mechanism as is the case under a zero-inflated model.

This study aimed to evaluate the utility of a Bayesian forecasting model to predict LACV human cases within the endemic Appalachian region, USA. A combination of traditional forecasting and leave-one-out forecasting were performed using an integrated nested Laplace approximation approach, a computationally efficient method. After variable selection, the model accurately predicted LACV cases in 97.72% of counties for the leave-one-out validation, and 98.08% for the traditional forecasting, with a maximum error of two cases. Most findings regarding the regression coefficients were consistent with the existing literature, adding rigor to this approach.

## 5. Conclusions

La Crosse virus is an important cause of encephalitis in children in the USA, and forecasting models can be used to identify areas of increased transmission risk, improving case detection. The INLA model is a fast, inexpensive, and efficient statistical tool that can be implemented to design targeted control or surveillance strategies. In order to ensure prediction models are accurate, efforts must be made in improving the accessibility of datasets and promoting surveillance to accurately detect cases. This technical study demonstrates the utility of advancing mathematical modelling for public health decision making and targeted intervention.

## Figures and Tables

**Table 1:** Statistically Significant Variable Estimation with Fixed Effects.

Variable	Estimate	Standard Deviation	Lower Bound of 95% HPD Interval	Upper Bound of 95% HPD Interval
Intercept	−3.7310	0.2201	−4.1707	−3.3098
Proportion Age 19 and under	0.4006	0.0768	0.2504	0.5515
Developed Open Space	0.8503	0.0818	0.6909	1.0116
Developed High Intensity	−0.3587	0.0819	−0.5199	−0.1986
Barren Land	0.1763	0.0577	0.0638	0.2901
Evergreen Forest	−0.4000	0.1488	−0.6938	−0.1105
Hay Pasture	−0.2931	0.0721	−0.4350	−0.1525
Woody Wetland	−3.3714	0.5984	−4.5671	−2.2267
Vapor Pressure Deficit index	−0.2669	0.0937	−0.4511	−0.0838
Year	−0.0391	0.0794	−0.1948	0.1162

## Data Availability

The data supporting reported results can be found in publicly archived datasets as follows: La Crosse Virus human case data was obtained from the U.S. Centers for Disease Control and Prevention ArboNET database website (url currently unavailable); Rodent density data was obtained from the United States Geological Survey website (https://gapanalysis.usgs.gov/apps/species-data-download/ (accessed on 6 January 2022)); Land cover data was obtained from the National Land Cover Database (https://www.usgs.gov/centers/eros/science/national-land-cover-database (accessed on 6 January 2022)); Weather data was obtained from the Northwest Alliance for Computational Science’s PRISM climate dataset (https://prism.oregonstate.edu/ (accessed on 6 January 2022)).

## Supplementary Materials

The following supporting information can be downloaded at: https://www.mdpi.com/article/10.3390/microorganisms13040812/s1, Table S1: Forecasting Model Mean Square Prediction Error of Leave-One-Out Prediction Validation and Counties with Highest Error, by Year; Table S2: Forecasting Model Mean Square Prediction Error and Counties with Highest Error, by Year; Figure S1: 95% Highest Posterior Distribution Lower and Upper Bounds for 2021 Predictions; Figure S2: Observed Case Counts, by Year.

## Abbreviations

The following abbreviations are used in this manuscript:

ACF	Autocorrelation factor
ARC	Appalachian Regional Commission
CDC	United States Centers for Disease Control and Prevention
INLA	Integrated nested Laplace approximation
LACV	La Crosse Virus
MCMC	Markov chain Monte Carlo
NLCD	National Land Cover Database
USA	United States of America
VIF	Variance inflation factor
VPD	Vapor pressure deficit
