# Supplementary material for: Validating a Bayesian Spatio-Temporal Model to Predict La Crosse Virus Human Incidence in the Appalachian Mountain Region, USA
Source: Microorganisms. 2025 Apr 3;13(4):812. doi: 10.3390/microorganisms13040812 (PMC12029143; doi:10.3390/microorganisms13040812)
Supplement: Supplementary file 1 [file microorganisms-13-00812-s001.zip › microorganisms-3540772-supplementary.pdf]

Validating a Bayesian Spatio-Temporal Model to Predict La Crosse Virus Human Incidence in the Appalachian Mountain Region, USA

Maggie McCarter [1], Stella C. W. Self [1], Huixuan Li [1], Joseph A. Ewing [2], Lídia Gual-Gonzalez [1], Mufaro Kanyangarara [1] and Melissa S. Nolan [1,*]

[1] Department of Epidemiology and Biostatistics, Arnold School of Public Health, University of South Carolina, 915 Greene Street, Columbia, SC 29208, USA; maggiemccarter@gmail.com (M.M.); scwatson@mailbox.sc.edu (S.C.W.S.); huixuan@email.sc.edu (H.L.); lidiag@email.sc.edu (L.G.-G.); mufaro@mailbox.sc.edu (M.K.)

[2] Data Support Core, Prisma Health, 701 Grove Rd, Greenville, SC 29605, USA; alex.ewing@prismahealth.org

* Correspondence: msnolan@mailbox.sc.edu

Table S1: Forecasting Model Mean Square Prediction Error of Leave-One-Out Prediction Validation and Counties with Highest Error, by Year

Year	Mean Square Prediction Error (Cases^2)	Counties with Error >= 2 Count
2010	0.4424	Forsyth, NC; Forsyth, GA; Buncombe, NC; Holmes, OH; Tuscarawa, OH; Kanawha, WV; Swain, NC; Transylvania, NC
2011	0.6203	Forsyth, NC; Forsyth, GA; Buncombe, NC; Knox, TN; Raleigh, WV; Transylvania, NC; Mercer, WV; Ross, OH, Muskingum, OH; Macon, NC;
2012	0.5704	Forsyth, NC; Forsyth, GA; Buncombe, NC; Knox, TN; Transylvania, NC
2013	0.2686	Forsyth, NC; Forsyth, GA; Knox, TN; Claiborne, TN; Raleigh, WV; Jackson, NC
2014	0.4095	Forsyth, NC; Forsyth, GA; Buncombe, NC; Jackson, NC; Knox, TN; Coshocton, OH; Muskingum, OH; Holmes, OH
2015	0.1753	Forsyth, NC; Forsyth, GA; Transylvania, NC; Knox. TN
2016	0.1050	Forsyth, NC; Forsyth, GA
2017	0.4035	Forsyth, NC; Forsyth, GA; Buncombe, NC; Jackson, NC; Cocke, TN; Grainger, TN; Knox, TN
2018	0.3751	Forsyth, NC; Forsyth, GA; Buncombe, NC; Knox, TN; Transylvania, NC; Haywood, NC; Jackson, NC; Raleigh, WV; Hocking, OH
2019	0.1628	Forsyth, NC; Knox, TN
2020	0.3725	Forsyth, NC; Holmes, OH; Jackson, NC; Buncombe, NC; Knox. TN; Transylvania, TN; Jefferson, TN
2021	0.0986	Forsyth, NC; Forsyth, GA

Table S2: Forecasting Model Mean Square Prediction Error and Counties with Highest Error, by Year

Year	Mean Square Prediction Error (Cases^2)	Counties with Error > 2 Count
2015	0.2759	Forsyth, NC; Forsyth, GA; Transylvania, NC; Knox, TN
2016	0.0740	Buncombe, NC
2017	0.3918	Buncombe, NC; Jackson, NC; Cocke, TN; Grainger, TN; Knox, TN
2018	0.3607	Forsyth, NC; Buncombe, NC; Knox, TN; Transylvania, NC; Haywood, NC; Jackson, NC; Raleigh, WV; Hocking, OH
2019	0.1553	Forsyth, NC; Knox, TN
2020	0.3715	Forsyth, NC; Holmes, OH; Jackson, NC; Buncombe, NC; Knox, TN; Jefferson, TN; Transylvania, NC
2021	0.0986	Forsyth, NC; Forsyth, GA

Figure S1: 95% Highest Posterior Distribution Lower and Upper Bounds for 2021 Predictions

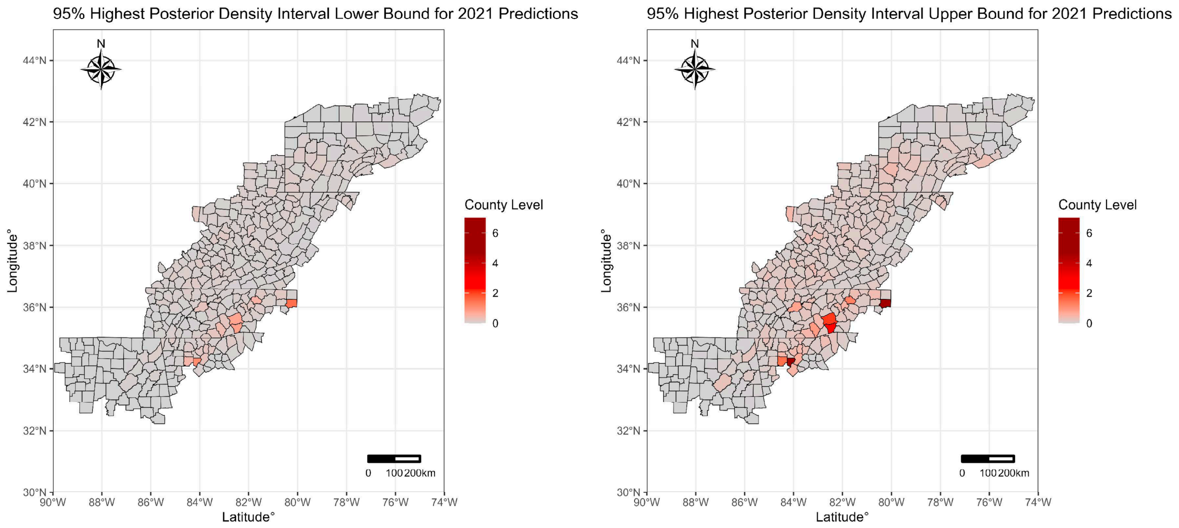

(a) (b)

Figure S2: Observed Case Counts, by Year

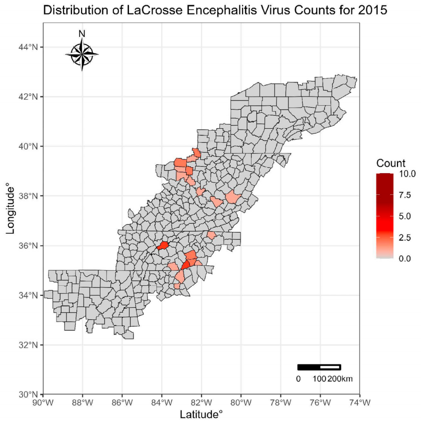

(c)

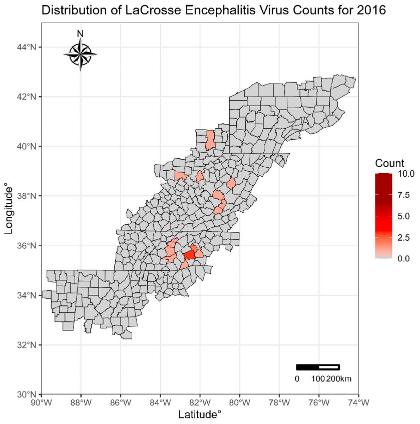

(d)

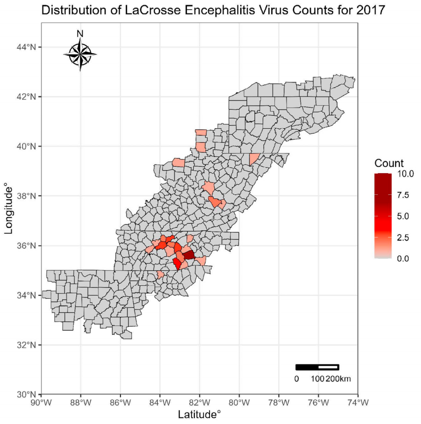

(e)

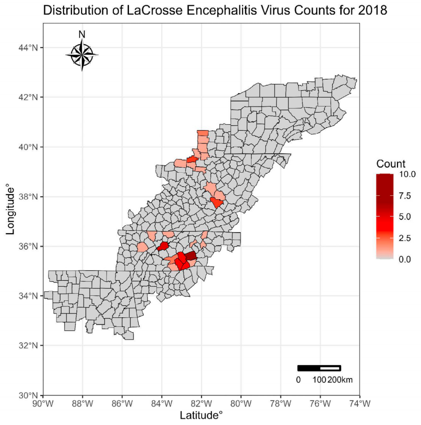

(f)

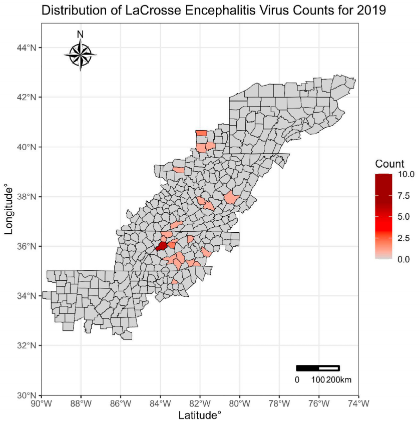

(g)

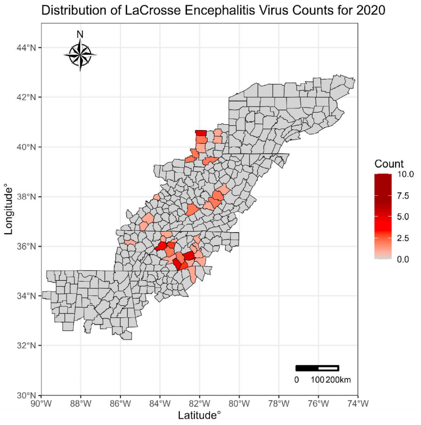

(h)
